# Deterioration of Basic Properties of the Materials in FRP-Strengthening RC Structures under Ultraviolet Exposure

**DOI:** 10.3390/polym9090402

**Published:** 2017-08-30

**Authors:** Jun Zhao, Gaochuang Cai, Lu Cui, Amir Si Larbi, Konstantinos Daniel Tsavdaridis

**Affiliations:** 1 School of Mechanics and Engineering Science, Zhengzhou University, Zhengzhou 450001, China; zhaoj@zzu.edu.cn; 2 School of Natural and Built Environment, Queen’s University Belfast, Belfast BT7 1NN, UK; 3 Ecole Nationale d’Ingénieurs de Saint-Etienne (ENISE), Laboratoire de Tribologie et de Dynamique des Systèmes (LTDS), Université de Lyon, UMR 5513, 58 rue Jean Parot, 42023 Saint-Etienne CEDEX 2, France; amir.si-larbi@enise.fr; 4 Multi-Functional Designs and Research Academy of Zhengzhou University, Zhengzhou 450001, China; a-vv@163.com; 5 School of Civil Engineering, University of Leeds, Leeds LS2 9JT, UK; K.Tsavdaridis@leeds.ac.uk

**Keywords:** ultraviolet exposure, fibre reinforced polymers strengthening, resin materials, ultimate tensile, concrete degradation

## Abstract

This paper presents an experimental study of the basic properties of the main materials found in reinforced concrete (RC) structures strengthened by fibre reinforced polymer (FRP) sheets with scope to investigate the effect of ultraviolet (UV) exposure on the degradation of FRP, resin adhesive materials and concrete. The comparison studies focused on the physical change and mechanical properties of FRP sheet, and resin adhesive materials and concrete before and after UV exposure. However, the degradation mechanisms of the materials under UV exposure were not analyzed. The results show that the ultimate tensile strength and modulus of FRP sheets decrease with UV exposure time and the main degradation of FRP-strengthened RC structures is dependent on the degradation of resin adhesive materials. The increase in the number of FRP layers cannot help to reduce the effect of UV exposure on the performance of these materials. However, it was verified that carbon FRP materials have a relatively stable strength and elastic modulus, and the improvement of the compression strength of concrete was also observed after UV exposure.

## 1. Introduction

With increasing environmental problems, civil engineers and researchers are concerned with the durability properties of reinforced concrete (RC) members. Compared with external post-tension strengthening—an accomplished repairing technology for damaged RC structures that usually involves the use of complex anchoring devices—the strengthening method using fibre reinforced polymer (FRP) sheets offers some unique advantages such as low self-weight, fast repairing and high strength. These properties make the method a widely suitable technology for the rehabilitation of damaged structures. A number of investigations and analyses have been performed on the FRP strengthened RC structures in the forms of experimental and numerical studies. These studies verified that the structures strengthened by FRP sheet materials exhibit good mechanical and deformation properties such as load-carrying capacity, stiffness, and crack-resistance etc.

Although FRP materials offer attractive properties to repair damaged RC structures, the effect of an aggressive environment on the durability and structural performance of these structures strengthened by FRP materials is not understood completely. The durability of FRP materials themselves in the environments is also one of the primary issues limiting the development of these materials in the application of some infrastructure. Out of these environmental impacts, UV radiation is potentially the most damaging to polymeric materials [1] used in FRP strengthening. In particular, the combined effect of moisture, ultraviolet (UV) exposure and loading on the durability and structural performance of FRP-strengthened RC structures is not well-documented [2]. Some strengthening methods using high-performance protection layers [3] or coating layers [4] have been proposed in the practical repair and strengthening of structures, however, there are many FRP repair materials used in strengthened RC structures which are still directly exposed under UV environment. To date, only a limited number of studies have been reported regarding the UV durability of the concrete structure strengthened by FRP.

UV exposure leads to surface oxidation due to different chemical mechanisms related to the resin type as investigated in previous studies [5,6]. Through using unidirectional Kevlar-49 epoxy laminates of varying thickness, Larsson [7] indicated that both fibres and resin were affected by UV light in an FRP composite, which results in a dangerous decrease in strength properties. Gu [8] reported that the tensile strength and deformation elongation of basalt FRP sheets (BFRP) degraded slightly as UV exposure time increased. Saud et al. [9] also presented similar research on the degradation of FRP via an accelerated test and explained that this degradation was caused by the reaction of UV with the bond impregnating material. However, UV exposure has a non-negligible influence on the bond between concrete and FRP materials [8,10,11]. Yang et al. [11] indicated that the degradation of resin caused by UV light is the primary reason for the loss of bond between concrete and FRP. For this, the bond failure of the specimens majorly focused on the concrete interface zone. Compared with the cases of carbon FRP (CFRP) materials, the bond behaviour between BFRP sheets and concrete is more susceptible to UV environment [8]. Hulatt et al. [12] considered that the degradation of FRP materials is attributed to the fact that UV lights destroy the typical covalent bond keys in the polymer in FRP materials, which changes the status of the polymer into a free radical status which then leads to material photo-degradation. Several investigations have previously reported that moisture content has a great effect on the UV radiation deterioration and resin materials (e.g., [13,14,15,16]). However, limited studies reported that UV exposure does not significantly influence the development of concrete strength [8] or FRP sheets’ elastic modulus [12]. These studies presented that the UV-ageing of composite materials is usually triggered or promoted because of the further oxidation reaction of resin adhesive. Compared with other types of FRP sheets, CFRP has a more stable capacity to resist the negative effect of UV light on the properties of FRP and FRP-strengthened RC structures [8,17,18].

Regarding the structural elements, Gu [8] studied six RC beams and 16 RC columns strengthened by Basalt FRP (BFRP) and his main results are summarized as (1) the flexural strength of the RC beams decreases as UV exposure time is decreased for the tensile and stability of BFRP; (2) the axial compression capacity of the RC columns strengthened by BFRP reduces with UV exposure time, expect for the ones strengthened by BFRP strips; (3) the axial deformation capacity and ductility of the columns are reduced after UV exposure; (4) UV exposure did not affect the failure mode of the strengthened RC columns. However, very limited studies [2,8,19] have been conducted to investigate the structural strength and deformability of RC elements strengthened by FRP.

Therefore, it is very important for the maintenance and assessment of RC structures to understand the changes of the mechanical behavior of FRP materials when exposed to environmental effects during their life cycle. These effects could include UV radiation, freeze–thaw cycles, high relative humidity, aqueous solution exposure, chemical agents, or the combinations of two or more of these conditions. Until now, most of the existing studies focused on FRP material itself, in particular for carbon FRP, and studied parameters are limited to strengthened RC structures in the literature.

**Figure 1:**
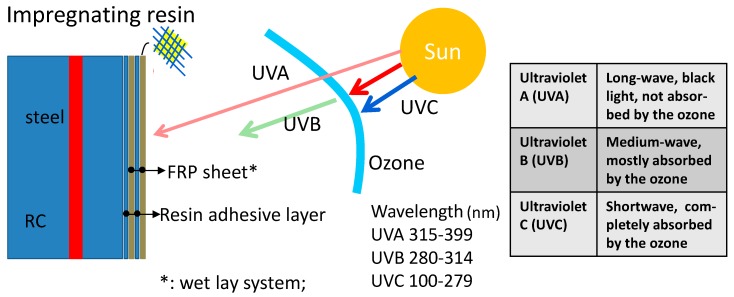
Potential effects on fibre reinforced polymer (FRP)-strengthened reinforced concrete (RC) structures from different UV lights.

In order to investigate the performance degradation of RC structures strengthened by various main FRP materials under ultraviolet light, a series of simulated accelerated test investigations have been performed, including studies on the deterioration of various FRP sheets themselves, impregnation resin and concrete under UV lights at the first stage. Figure 1 shows the potential deterioration actions caused by UV lights in the concrete structures strengthened by FRP materials, and the available types of UV lights from the sun. The objective of the current study is therefore to experimentally investigate the deterioration mechanism and the resultant changes in the mechanical properties of the materials involved when exposed to ultraviolet radiation.

## 2. Materials and Methods

**Figure 2:**
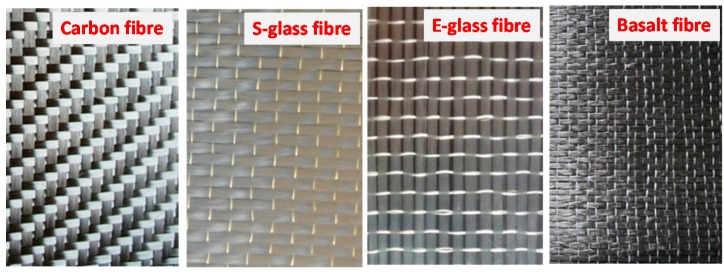
FRP sheet materials used in the study.

The FRP sheet materials applied in the study have four types, carbon FRP (CFRP), Basalt FRP (BFRP), E-type Glass FRP (EGFRP), and S-type Glass FRP (SGFRP), as shown in Figure 2. All FRP sheets were fabricated through a pultrusion process by containing these fibre materials. The detailed mechanical properties of these FRP sheets provided by their manufacturers are listed in Table 1. These various FRPs were used in order to study the effect of UV lights on FRP-strengthened RC systems. Meanwhile, three different resin and adhesive materials were applied herein. Resins No. 1 to No. 3 in this study represent a combined adhesive material consisting of vinyl ester based resins (VERs), epoxy resins (ERs) and vinyl ester-epoxy based resins (VEERs) and three polyamide-curing agents (PCAs) from different manufacturers, respectively. Table 2 shows the main properties of the various resin and adhesive materials.

### 2.1. Preparation of the Specimens

**Figure 3:**
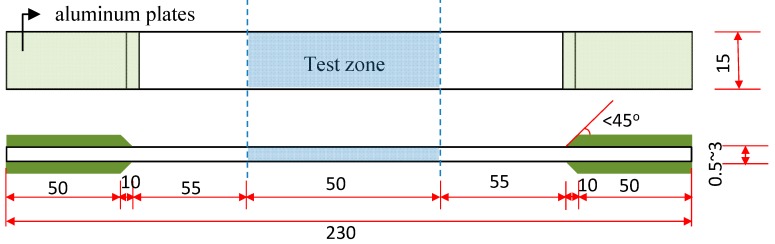
Details of FRP sheet specimens (in mm).

With regards to the test of the FRP sheets, a total of 648 sheet specimens have been investigated in this paper. The main parameters considered are resin type, UV exposure time and the type of FRP sheets. According to the Chinese standard test method, JG/T 167-2004—Carbon fibre laminate for strengthening and restoring structures [20], each FRC sheet specimen has a total length of 230 mm and a width of 15 mm. The test zone is designed as the middle part of the specimens with a length of 50 mm, as shown in Figure 3. Depending on the type of the FRP and number of layers of the FRP sheet, the thickness of these specimens varies from 0.5 mm to 3 mm. To reduce the stress concentration at the ends of the specimen, two strengthening ends were used in each specimen consisting of four stiff aluminum plates with a thickness of greater than 2.0 mm. The internal ends of the plates were grounded into a slope with an angle of less than 45 degrees as shown in Figure 3. In order to measure effective test results, six specimens were conducted for investigating each parameter. The detailed dimensions of the specimens are presented in Figure 3.

**Figure 4:**
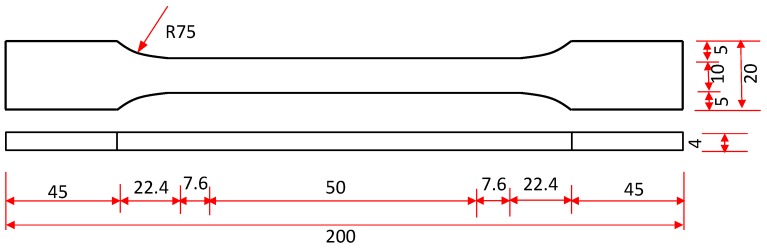
Details of resin plate specimens (in mm).

In order to investigate the strength and elastic modulus of resin materials, in this study, some dog-bone type specimens were manufactured as per Chinese code GB/T 2567-2008 [21]. As shown in Figure 4, each specimen is a long plate with a total length of 200 mm and a thickness of 4.0 mm. The test zone is the middle zone of the specimens with a length of 50 mm and a width of 10 mm. Two ends were set to have a loading buffer for each specimen, which both have a length of 45 mm and a width of 20 mm. Before they were put in the UV room, the resin specimens were placed at their natural temperature for 7 days.

Three 100 mm × 100 mm × 100 mm concrete cubes were cast to study the deterioration of the compressive strength of the concretes with each parameter subjected to UV exposure effects. Three different concrete strength grades were employed for this study with details given in Table 3. To construct all specimens, natural crushed limestone with a maximum diameter of 10 mm (nominal sieve size) and natural river sands with a fineness modulus of 2.6 were used as coarse and fine aggregate, respectively. All tested concrete specimens were first cured in a moist chamber with the relative humidity of 90–95% and temperature of 18–22 °C for 28 days before being subjected to UV exposure. It should be noted that the UV exposure time of the concrete did not include their curing days in the moist chamber.

### 2.2. Test Methods

**Figure 5:**
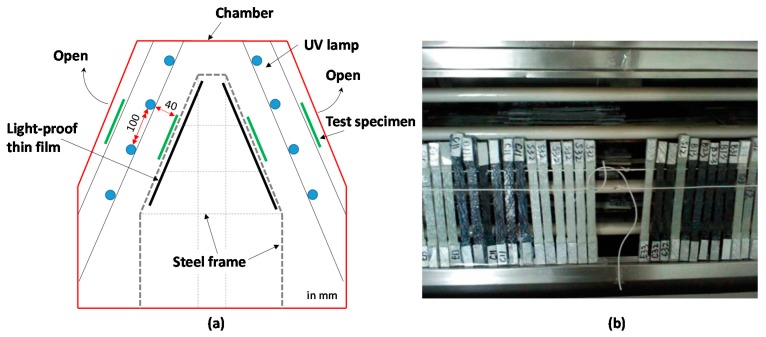
UV test chamber: (**a**) cross section and (**b**) arrangement of samples.

Regarding UV accelerated exposure testing, referring to international standards ISO 4892-3:1994 [22], a series of accelerated tests was performed to simulate the effect of UV exposure on FRP sheets and other related materials. Eight UV lamps were uniformly distributed in a UV test chamber and monitored carefully during the test, which provides stable and continuous UV lights with a wavelength ranging from 280 mm to 315 mm. The specimens were placed on a steel frame with certain spacing; only one surface of which faces the UV light directly. On the other side of the specimens, a black color and a light-proof plastic thin film were used to simulate the inside face of FRP sheets in repaired RC structures. All UV exposure tests were conducted in a cyclic way consisting of an 8 h UV exposure and a 4 h condensation process. Figure 5 shows the UV test chamber and the arrangement of the FRP samples in the UV chamber.

In this paper, the FRP-strengthened structures were assumed to be under the most severe natural moisture and temperature conditions (i.e., humidity is about 95% and the temperature is about 60 °C), and focused on the UV exposure of the main materials in such strengthened structures. Therefore, the UV test chamber was monitored through some smart sensors for keeping a stable UV environment for the specimens with a humidity ranging 90% to 95% and a temperature spanning from 57 °C to 63 °C. The opening of the UV test chamber was minimized for removing test samples to reduce the effects of the outside environment. All specimens were placed at room temperature for 48 h to eliminate possible residual stress on the specimen surface before testing them. All FRP and resin specimens were tested under an axial tension loading at a deformation rate of 2 mm/s, as per the Chinese test standard [21]. An external extensometer was used to measure the deformation of the test samples. The effect of UV on the basic properties of concrete is also investigated via the standard compressive strength test, and a scanning electron microscope (SEM) and X-ray diffraction (XRD) were used to study changes to the microstructural and hydration products of all of the concrete samples.

## 3. Results and Discussions

In this section, with the consideration of a large volume of experimental samples, the main results of the study are presented and discussed via analyzing and comparing some representative samples.

### 3.1. FRP Sheet Specimens

#### 3.1.1. Physical Changes

**Figure 6:**
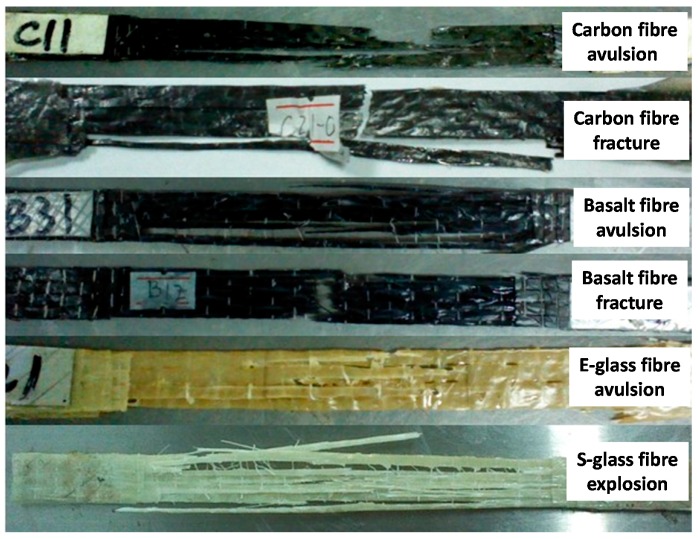
Typical failure situation of different FRP sheet specimens.

After 30 days of UV exposure, the surface colour of all tested EGFRP and SGFRP sheet specimens changed to yellow or deep yellow and exhibited no visual surface cracks. However, the cases of the CFRP and BFRP specimens are more stable, i.e., no obvious colour change and cracking. Figure 6 shows the detailed failure situation of different FRP materials. Most of the tested CFRP, BFRP, and EGFRP sheet specimens failed in fracture mode, while all SGFRP specimens fractured with a blasting failure or explosion damage.

#### 3.1.2. Effect of Variables on Mechanical Properties

**Figure 7:**
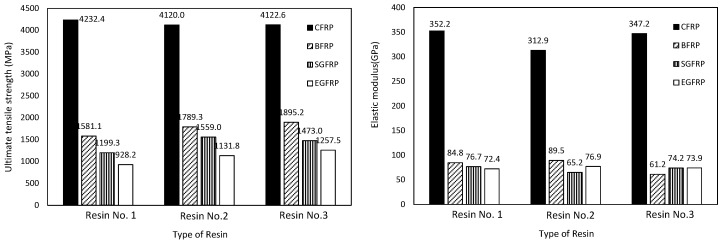
Tensile strength and elastic modulus of FRPs using different resin matrixes (without UV effect).

The results presented in Figure 7 show that CFRP sheet has a higher tensile strength and elastic modulus than other FRP specimens, regardless of the type of resin matrixes used. With regards to the effect of different resin matrixes, CFRP sheet that used Resin No. 2 has the smallest tensile strength and elastic modulus compared with the CFRP specimens in other resins. Although the elastic modulus of BFRP sheet using resin No. 3 is much less than the ones of the BFRP using the other two resins, the specimen has relatively high ultimate tensile strength. The type of resin matrix has no significant influence on the ultimate tensile strengths and elastic modulus of EGFRP and SGFRP sheets.

**Figure 8:**
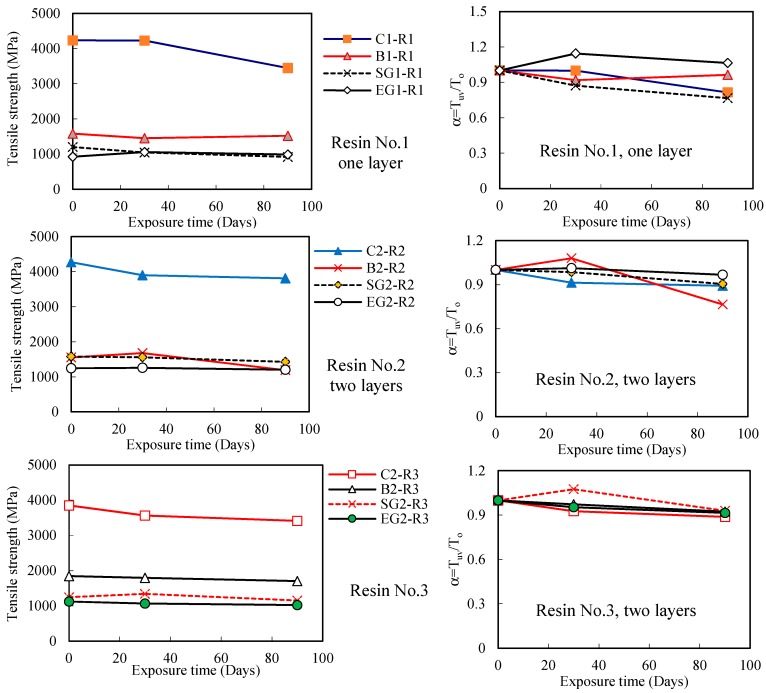
Effect of UV exposure on the tensile strength of different FRPs.

Regarding the effect of UV exposure time, when using Resin No. 1, the tensile strengths of all FRP sheets were affected by the UV exposure time, i.e., tensile strength reduced with the UV exposure time as shown in Figure 8. The tensile strengths of BFRP, SGFRP and EGFRP sheet specimens in the Resins No. 2 or No. 3, all present a similar result. For CFRP sheet using Resin No. 3, however, there is not any obvious degradation after 90 days of UV exposure. This does not mean that the FRP sheets are unaffected by UV environment, and implies that more investigations or longer exposure time tests need to be performed to further study these FRP sheets. Additionally, the results presented in Figure 8 show that tensile strength of CFRP is higher than that of other FRP sheets regardless of the type of resin materials they used.

**Figure 9:**
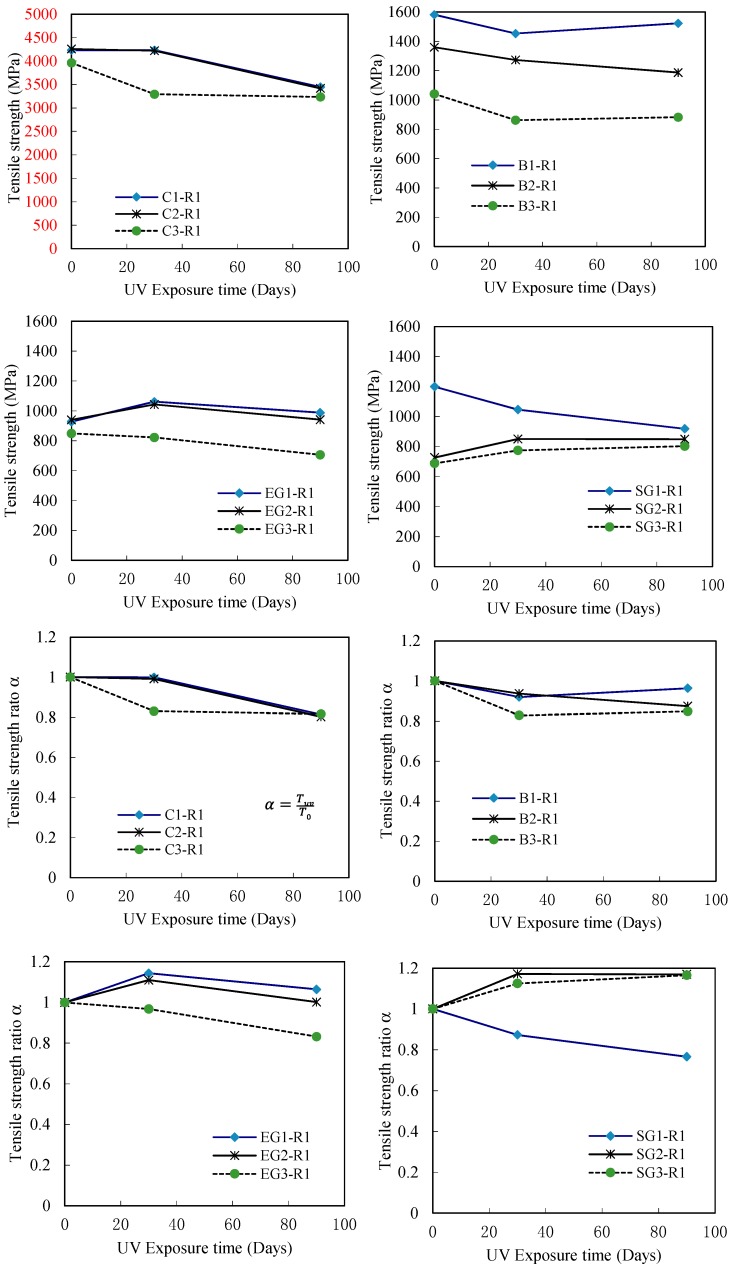
Effect of UV exposure on the tensile strength of FRP sheets with different layers (Resin No. 1).

**Figure 10:**
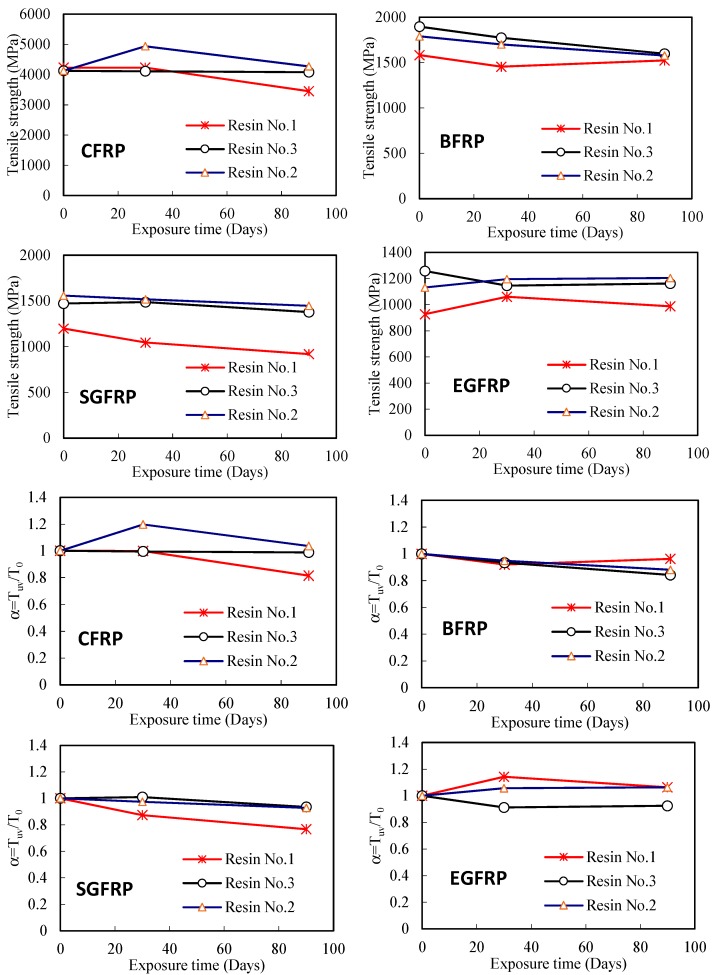
Effect of UV exposure on the ultimate tensile strength of FRP sheets using different resins (one layer).

Figure 9 shows the effect of UV exposure on the tensile strength of different FRP sheets with a different number of FRP layers, by presenting some series of represented specimens using Resin No. 1. In this figure, the specimens C1-R1, C2-R1 and C3-R1 represent the one, two and three layers of CFRP sheet specimens respectively, while the similar naming rule was applied in other FRP specimens as shown in Figure 9. The result confirms that the tensile strength of CFRP sheet decreased with the increase of the number of FRP layers, while the other FRP specimens have similar results with regards to UV exposure. Due to the external loading action which is transferred via the resin adhesive layers between FRP sheets, the un-uniform stress transferring between the resin adhesive and FRP sheet increases when the number of FRP sheet layers increases, which further results in a decrease in strength and elastic modulus of the test specimen. However, there is some unexpected increasing tensile strength of some specimens, which is explained by a result of the short hardening of combined resin adhesive materials in these specimens. Therefore, Figure 9 also shows the tensile strength ratio of these specimens. The results show that tensile strength decreases for most specimens as UV exposure time increases. On the other hand, the results plotted in Figure 10 show that the tensile strength of most one-layer FRP sheet specimens decreases as UV exposure time increases regardless of the resin adhesive material used, which is similar to the results in Figure 9. The results of the tensile strength ratio of the specimens in Figure 10 show a clear degradation in the tensile strengths of the FRP specimens.

It should be noted that the mechanical properties of FRP materials subjected to UV exposure are improved in some cases as shown in Figure 8, Figure 9 and Figure 10. This may be attributed to the low-degree volumetric shrinkage or the hardening of resin adhesive materials at an early stage [23] which may improve the overall mechanical characteristics of the combined resin adhesive and FRP. At that moment, the main intermolecular bonding energy is from the internal chemical bond energy, which is relatively stable. With increased UV exposure, more intermolecular bonding energy is destroyed leading to a decrease in the strength and module of these FRP sheets.

### 3.2. Resin Adhesive Materials

**Figure 11:**
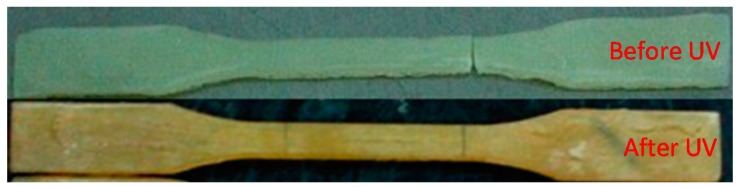
Comparison of physical changes of resin adhesive specimens before and after UV exposure.

Compared with the results of FRP specimens, the physical changing of resin adhesive specimens after UV exposure is remarkably larger. Figure 11 shows an obvious color change as observed in the tested specimens of Resin No. 1. In all specimens after UV exposure, obvious surface softening was observed before condensation at a natural temperature—some of them can even be elongated by hand.

#### 3.2.1. Effect of Variables on Mechanical Properties of Resin Adhesive Materials

**Figure 12:**
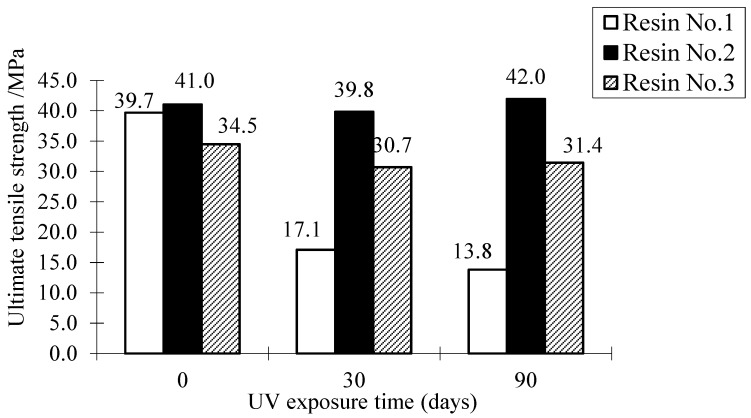
Effect of UV exposure on the ultimate tensile strength of resin.

**Figure 13:**
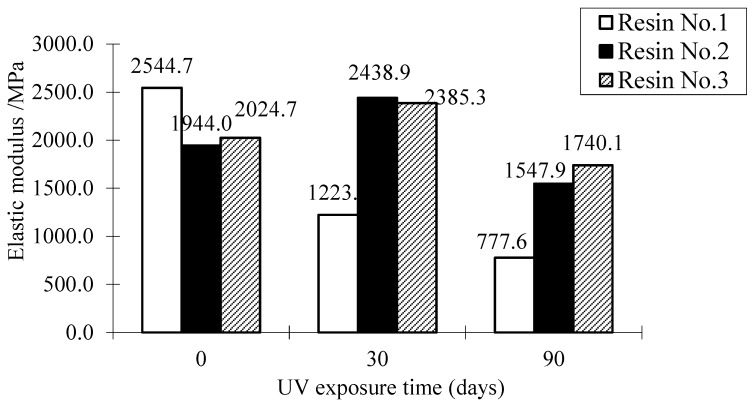
Effect of UV exposure on the elastic modulus of resin 3.3. Concrete degradation after UV exposure.

Figure 12 and Figure 13 show the effect of UV exposure on the ultimate tensile strength and elastic modulus of resin adhesive materials. The result shows that UV exposure has a significant effect on the tensile strength and elastic modulus of the resin No. 1. For instance, after the specimen was exposed to UV for 30 days, the tensile strength and elastic modulus of Resin No. 1 are 43% and 48% of the specimens without the UV effect. Moreover, the two properties of the same resin adhesive materials after 90 days of UV exposure continuously reduce their values from 35% and 31% when they are without UV exposure, respectively. The resin No. 3 shows a similar trend after UV exposure for 30 and 90 days. Compared with the other two types, although its elastic modulus also reduces after long-term (90 days) UV exposure, the resin No. 2 has a stable strength trend with UV exposure (i.e., it lost only 5% of its tensile strength when resin underwent 30 days of UV exposure). On the other hand, although the elastic modulus of resin No. 2 and No. 3 both reduce the long-term UV exposure as shown in Figure 13, their values both increase when they are subjected to 30 days UV exposure. This trend explains the transitory increase of tensile strength of FRP when using resin No. 2 in the above results (see the cases in Figure 7, Figure 8, Figure9 and Figure 10). As discussed previously, this is because of the low-degree volumetric shrinkage [23] or hardening and internal status change of the resin matrix at an early stage and the main intermolecular bonding energy in epoxy resin which is relatively stable and is not easily destroyed [24]. On the other hand, Resin No. 2 is epoxy-based resin, the mechanical properties of which are usually improved after UV exposure [25], which partly explains the initial increase of the tensile strength and elastic modulus of the resin. For resin No. 1, one kind of vinyl ester resin, its mechanical property is affected by UV exposure significantly [26], and the ultimate tensile strength and elastic modulus of the resin adhesive materials decreased with UV exposure, even at the early stage. The mechanical changes in Resin No. 3 are milder and are highly unstable due to the combined resin adhesive using vinyl ester-epoxy based resins whose properties are between the ones of vinyl ester resin and epoxy resin.

#### 3.2.2. Concrete Compressive Strength

**Figure 14:**
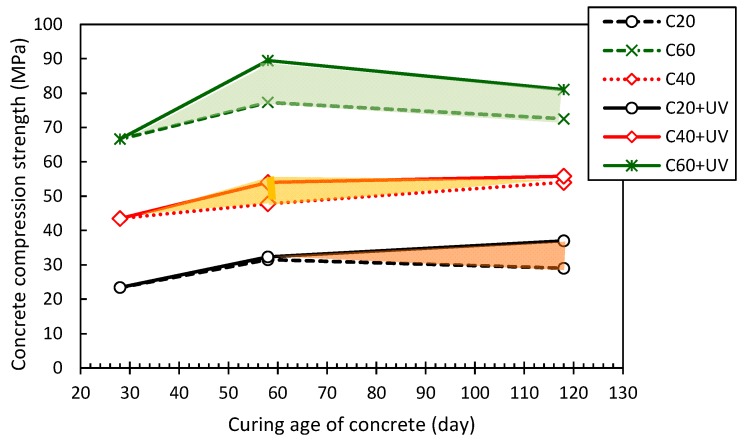
Effect of UV exposure on concrete compressive strength.

Figure 14 shows that UV exposure has a significant influence on the compressive strength development of concrete, in particular for low strength concretes, which have strength enhancement ratios of 38.2% and 58.3% in the concretes that were subjected to UV exposure for 30 days and 90 days, respectively. However, this enhancement may come from the self-strength development of the concretes under high temperature and moisture environment in the UV exposure chamber. Therefore, more detailed SEM and XRD analyses were conducted to investigate the micro-structural and chemical changes of the concretes subjected to UV exposure. Owing to the use of a water-reducing agent in the concrete C60, the agent may have an advantageous effect on the early-stage strength of the concrete, leading to a decrease in the later strength of the concrete at 118 days (UV exposure of 90 days).

#### 3.2.3. SEM Analysis-Microstructural Development of Concrete

**Figure 15:**
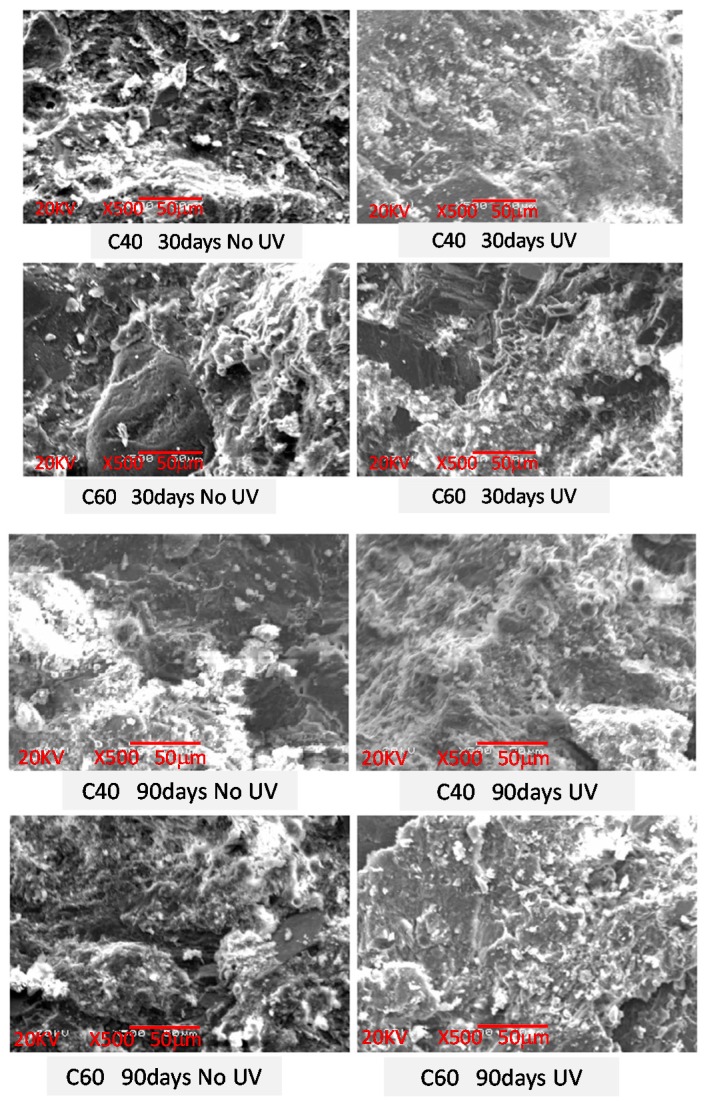
SEM comparison of concrete before and after UV exposure.

Figure 15 shows the comparison between the SEM results of some representative concrete specimens before and after the UV exposure. Without further explanation, this is not obvious with almost identical micro-structures being seen in all instances. Meanwhile, no obvious needle-like Ettringite and flocculent C-S-H were observed in all SEM pictures, which means that these Ettringite and C-S-H productions already interacted in a compact structure which contributes to the strength development of concrete. However, as the UV tests are done in a high humidity environment, the increase in the microstructure compactness may happen as more cement hydration, due to UV exposure, elevates the temperature which makes more water permeate the internal concrete via the capillary. Therefore, the increase in strength of the concretes after UV exposure may be attributed to the physical improvement of concrete microstructures. It is suggested that further study on this aspect is needed.

#### 3.2.4. XRD Analysis-Hydration Effect of Concrete

**Figure 16:**
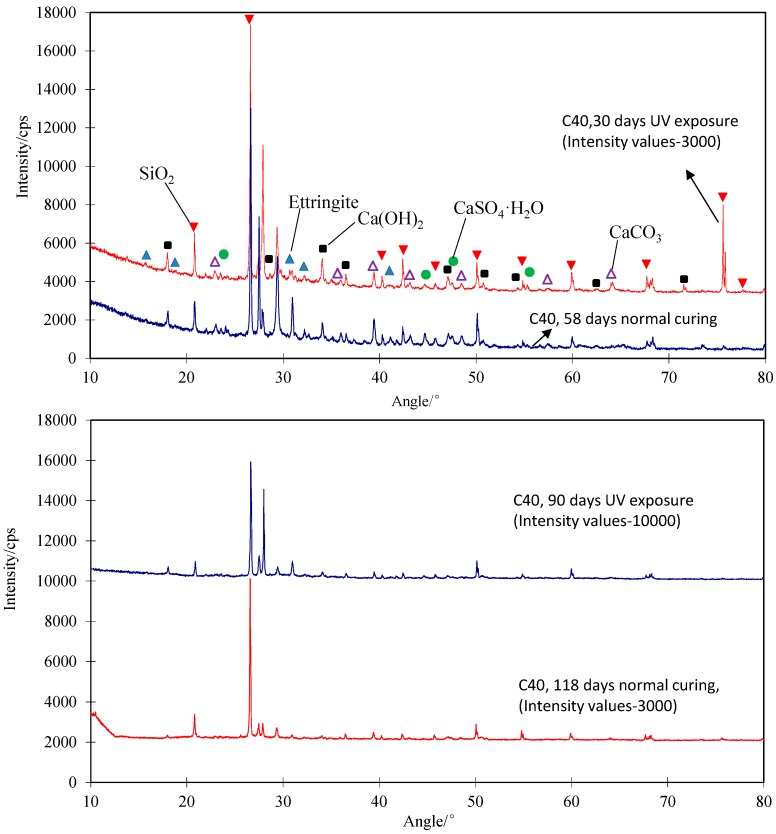
X-ray diffraction analysis of concrete (40 MPa) before and after UV exposure.

As shown in Figure 16, the main chemical compositions of the concrete without and with 30 days of UV exposure are SiO_2_ and Ettringite, respectively. In the concretes having 90 days normal curing without the UV effects (i.e., a total of 118 days normal curing), there is a high content of CaSO_4_·H_2_O compared with the ones in the concrete with 30 days normal curing (i.e., a total of 58 days normal curing). On the other hand, similar to the results plotted in Figure 16, in other cases such as the low strength concrete specimens (20 MPa), the concrete contains more CaSO_4_·H_2_O and Ca(OH)_2_ after the 30 days normal curing as well as more Ettringite and Ca(OH)_2_ after 30 days UV exposure. In the high strength concrete (60 MPa), a similar result was observed. However, as described above, the changes to the hydration products in concrete are possibly due to more hydration in concrete for the entry of more water because of the combined effects of a highly humid external environment and UV exposure. Therefore, the results implied that UV exposure might affect the chemical compositions of concretes that contribute to their compressive strength development. Further research on this aspect is needed.

## 4. Deterioration Mechanism

**Figure 17:**
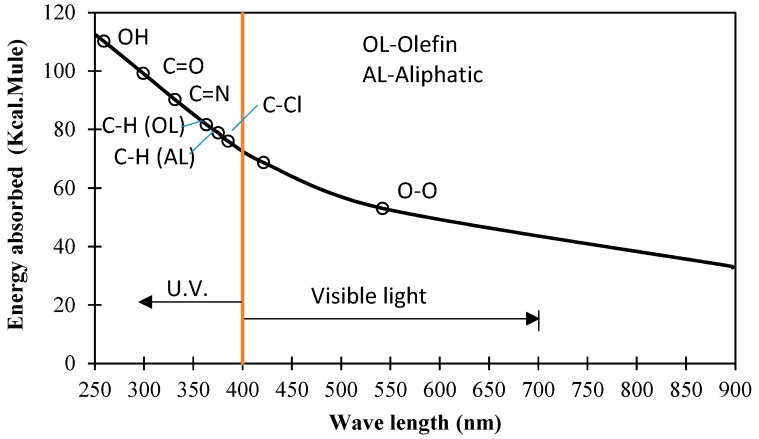
Representative chemical bond keys and their absorption energies.

**Figure 18:**
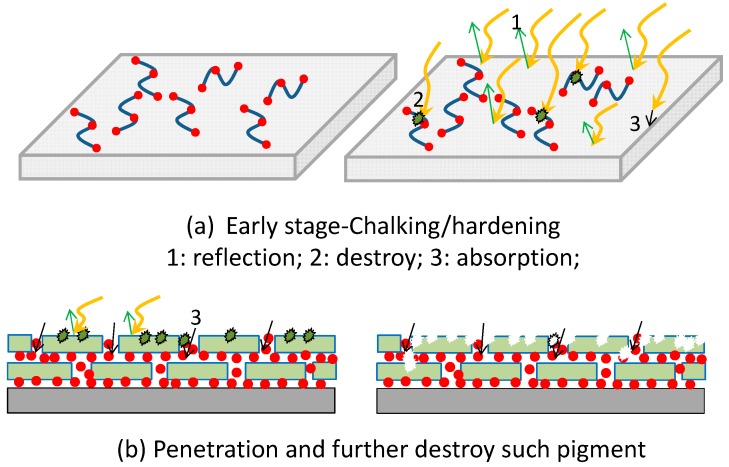
Deterioration mechanism of FRP materials caused by UV exposure.

The main reason for the destruction of polymer molecular bonds under UV exposure is their intermolecular bonding energy, which is lower than that of UV light. Figure 17 shows the potentially affected polymer molecular bonds in FRP materials and their related materials such as resin adhesive materials when they are subjected to UV exposure. Therefore, the degradation mechanisms of FRP materials under UV exposure are summarized as follows:

- When the fibre composite materials are irradiated by UV radiation, the resin adhesive materials on their surface firstly contact the radiation waves, and the resin molecules absorb the high-energy light wave to produce the photo-aging reaction which makes the materials turn yellow and leads to cracking. However, at the early stage, the overall mechanical characteristics of some FRP specimens may also be improved due to the low-degree volumetric shrinkage or the hardening of resin adhesive materials at the early stage. It is also possible that the main intermolecular bonding energy in some resins is from some stable chemical bonds (Figure 18a).
- As the wavelength of ultraviolet light is small and quite poor in its penetrative ability, most of the radiant energy can only affect the surface of resin adhesive materials. Only a small amount of longer radiation energy of the ultraviolet radiation can destroy the surface, so the damage of fibre reinforced materials is much lower than the damage to the resin materials. However, as shown in Figure 18a, the chalking of resin happened on the surface and the cracking was extended.
- With UV exposure, for more UV lights to enter and touch FRP sheet, a large number of molecular bonds were then destroyed, which leads to decreases in the strength and module of these FRP sheets. The reason for the decreased strength of FRP material after ultraviolet radiation is that the capacity and effectiveness of the stress transmission between FRP materials or between resin adhesive and FRPs are reduced, which is caused by the further decrease in the strength and modulus of resin adhesive materials or its pulverization due to longer UV radiation exposure (Figure 18b).
- In a typical strengthening of the damaged RC structure, limited UV lights can reach the concrete–FRP interface zone; the effect of UV exposure on the bond degradation between FRP and concrete should be theoretically very small. More detailed studies should be conducted in the future to evaluate the effect of UV exposure on the bond degradation between FRP, resin adhesive layer and concrete.

## 5. Concluding Remarks

Through a series of experimental studies, this paper investigates the changes in the mechanical properties of the main materials in RC structures strengthened by FRP sheets under UV exposure, while mainly focusing on the degradation of the FRP performance, resin adhesive materials and concrete after UV exposure. The main conclusions are drawn as follows:

**For FRP sheets under UV exposure:**

- UV exposure has a different influence on the basic properties of different FRP sheets. Based on this study, among the four types of FRP sheets, CFRP has the most stable mechanical properties after UV exposure. The basic properties of BFRP were affected significantly by UV exposure and are lower than those of other FRP sheets.
- Based on this study, the increase of the number of FRP layers cannot reduce the effect of UV exposure on the basic mechanical behavior of FRP sheets, which is partly attributed to the instability between the FRPs and adhesive layers. However, more research on the number of FRP layers on the UV effect in the FRP–concrete interface zone, such as the bond behavior, is expected in the future.
- The material properties of adhesive can effectively determine the FRP-strengthening of RC structures through affecting the degradation of FRP sheets. The degradation of the mechanical properties of FRP sheets due to UV exposure can be different when different adhesive materials are used.
- In the initial stage, the mechanical properties of some FRP sheet specimens are improved which is due to the low-degree volumetric shrinkage or hardening and internal status change of resin adhesive material, improving the mechanical characteristics of the resin adhesive and FRP. However, the main intermolecular bonds in the resins at the early stage are more stable and are also one of the reasons for the improvement.

**For resin adhesive materials under UV exposure:**

- The effects of UV exposure on the tensile strength and module properties of different resin adhesive materials are different. Additionally, the study implied that the UV exposure time leading to the property degradation is different when different resin materials were used.
- FRP can provide a reinforcement action to the properties of resin adhesive materials due to the effect of UV exposure starting from the surface of resin adhesive materials.

**For concrete under UV exposure:**

- The compressive strength of concrete was improved after UV exposure in the chamber having a certain humidity and temperature, which indicated that it may be helpful to concrete strength development when the concrete is under the three environmental effects.
- Based on the SEM and XRD analyses, after UV exposure, the physical microstructure of concrete was improved and more strength-favorable chemical compositions were produced in the concrete.

Based on the experimental studies, this paper explained the deterioration mechanism of the basic mechanical properties of the main materials related to the FRP-strengthened RC structures. The main processes were simply concluded as surface deterioration such as hardening or chalking/ageing, UV absorption and penetration, and the destruction of internal bond-energy.

## Figures and Tables

**Table 1:** Mechanical properties of used fibre reinforced polymer (FRP) sheets (provided by the manufacturer).

Properties	Carbon FRP (CFRP)	Basalt FRP (BFRP)	S-Type Glass FRP (SGFRP)	E-Type Glass FRP (EGFRP)
Tensile strength/MPa	3905	2335	2279	1620
Tensile elastic modulus/GPa	228	105	106	81.4
Ultimate strain/%	1.67	2.1	2.76	1.49
Nominal thickness/mm	0.111	0.107	0.2	0.122

**Table 2:** Properties of three combined adhesives.

Properties	Units	Resin No. 1	Resin No. 2	Resin No. 3
		VERs + PCAs	Ers + PCAs	VEERs + PCAs
Ultimate Tensile strength	MPa	48.6	46.4	44.3
Elastic modulus	MPa	2611	2620	2598
Ultimate strain	%	1.58	1.62	1.66
Flexural strength	MPa	72.4	68.3	71.2
Compressive strength	MPa	86	76.7	74
Bond strength (with concrete) ^1^	MPa	4.6	3.4	3.9
Shear strength (steel/steel, tensile)	MPa	18.9	15.9	16.8
Solid weight ratio	%	99.4	99.4	99.4
Check per Chinese code	-	ok	ok	ok

^1^ The values are obtained via an axial tensile test until concrete failure.

**Table 3:** Details of concrete mix.

No.	w/c ^1^	Water (kg/m^3^)	Cement (kg/m^3^)	Sands (kg/m^3^)	Gravels (kg/m^3^)	WRA ^2^ (kg/m^3^)	UV Exposure Time (After 28 Days in Moist Chamber)
C20	0.6	180	300	689.5	1280.5	0	30/60/90
C40	0.45	180	400	654.5	1215.5	0	
C60	0.31	180	580	591.5	1098.5	11.6 ^3^	

^1^ Water/cement ratio; ^2^ Water reducing admixture; ^3^ 2% of cement mass.
